# Mechanism of Astragaloside IV Against Cerebral Ischemia–Reperfusion Injury: Inhibiting Neuronal Apoptosis via the CytC/Apaf-1 Mitochondrial Pathway

**DOI:** 10.3390/ph19040547

**Published:** 2026-03-29

**Authors:** Tongtong He, Zhe Zhang, Xiaohong Zhou, Ping Gao, Zhenyi Liu, Yanmeng Zhao, Hua Liang, Weijuan Gao, Xiaofei Jin

**Affiliations:** 1 Hebei Key Laboratory of Chinese Medicine Research on Cardio-Cerebrovascular Disease, Hebei University of Chinese Medicine, No. 3 Xingyuan Road, Luquan District, Shijiazhuang 050200, China; halona0211@hebcm.edu.cn (T.H.); zhouxiaohong@hebcm.edu.cn (X.Z.); gaoping0321@163.com (P.G.); jiushihenhaoji@126.com (Z.L.); zhaoyanmeng@hebcm.edu.cn (Y.Z.); lh1701100040@hebcm.edu.cn (H.L.); 2 Traditional Chinese Medicine Program, Graduate School, Juquan Campus, Hebei University of Chinese Medicine, No. 326, Xinshinan Road, Qiaoxi District, Shijiazhuang 050091, China; m15233410887@163.com

**Keywords:** astragaloside IV, cerebral ischemic reperfusion injury, CytC/Apaf-1 pathway, neuron apoptosis, molecular dynamics simulation

## Abstract

**Background:** Neuronal apoptosis is the core pathological mechanism of cerebral ischemic–reperfusion injury (CIRI); although Astragaloside IV (AS-IV) has demonstrated neuroprotective activity against CIRI, its specific molecular mechanisms underlying the regulation of this apoptosis-related pathway remain to be systematically elucidated. **Methods:** We establish an in vivo model of middle cerebral artery occlusion/reperfusion (MCAO/R) in rats and an in vitro model of oxygen–glucose deprivation/reperfusion (OGD/R) in PC12 cells. Six core apoptotic proteins, including CytC, Apaf-1, BAX, Bcl-2, Caspase3, and Caspase9, were detected using neurological function scoring, TTC/HE/Nissl staining, TUNEL staining, Western blot, and immunofluorescence techniques. Molecular docking and molecular dynamics simulation were utilized to analyze the binding affinity between AS-IV and the aforementioned apoptotic proteins. **Results:** Molecular docking and dynamics simulation demonstrated AS-IV stably binds six core apoptotic proteins, and comparative analysis with target-specific reference ligands identified Apaf-1 as its primary target with the most favorable binding properties. In rat MCAO/R models, AS-IV alleviated neurological deficits, reduced cerebral infarct volume and improved brain pathological damage; in PC12 cell OGD/R models, it decreased neuronal apoptosis. Western blot and immunofluorescence confirmed AS-IV downregulated pro-apoptotic proteins (cytoplasmic CytC, Apaf-1, BAX, cleaved-Caspase9/3) and upregulated anti-apoptotic Bcl-2. **Conclusions:** This study clarifies the anti-apoptotic molecular mechanism of AS-IV, it alleviates CIRI by targeting the CytC/Apaf-1 mitochondrial apoptotic pathway.

## 1. Introduction

Cerebral ischemic–reperfusion injury (CIRI) is the core pathological process of secondary brain injury following vascular recanalization for ischemic stroke, with a complex pathogenesis involving mitochondrial dysfunction, neuronal apoptosis, oxidative stress outburst, unregulated inflammatory responses, and blood–brain barrier disruption via the synergistic action of multiple pathways [1,2,3,4]. Clinically, recombinant tissue plasminogen activator (rt-PA) thrombolysis and mechanical thrombectomy are the main strategies to restore blood perfusion in ischemic brain tissue. However, the narrow therapeutic time window, reperfusion-induced secondary injury, and limited repair capacity of damaged neurons severely hinder the improvement of prognosis in ischemic stroke patients, leaving unmet clinical therapeutic needs [5]. Thus, exploring the key regulatory pathways of CIRI and screening endogenous protective molecules for targeted intervention has become a major focus and urgent clinical need in ischemic stroke research.

Neuronal apoptosis is a core pathological event driving irreversible brain tissue damage and impairing neurological function recovery during CIRI progression [6]. Cerebral ischemia induces tissue hypoxia and intracellular microenvironment disturbances, which activate various pro-apoptotic signals [7,8]; reperfusion then triggers oxidative stress, calcium overload, and inflammatory factor infiltration, further amplifying the apoptotic signaling cascade and driving massive neuronal programmed cell death [9]. CIRI-induced neuronal apoptosis exhibits distinct time-dependent and spatial characteristics: ischemia-mediated apoptosis predominates in the early reperfusion stage [10], while inflammation-driven secondary apoptosis is prominent in the late stage, with apoptotic regions expanding from the ischemic core to the penumbra and directly determining the final extent of brain damage [11]. Notably, neurons in the ischemic penumbra undergo reversible injury, and effective inhibition of their apoptosis can significantly reduce cerebral infarction volume and improve neurological prognosis. Therefore, elucidating the key regulatory mechanisms of neuronal apoptosis in CIRI is a critical breakthrough for developing efficient neuroprotective strategies.

As the core regulatory axis of the mitochondrial apoptotic pathway, the CytC/Apaf-1 pathway plays a decisive role in CIRI-induced neuronal apoptosis [12,13]. Mitochondrial-released CytC binds to Apoptotic Protease Activating Factor-1 (Apaf-1) and ATP/dATP in the cytoplasm, inducing a conformational change in Apaf-1 to expose its N-terminal CARD. This domain then recruits cytoplasmic procaspase-9 via CARD-CARD interaction to form the CytC-Apaf-1-procaspase-9 apoptosome [14], which rapidly activates procaspase-9. The activated caspase-9 further triggers a downstream cascade reaction to activate effector caspases (caspase-3, -6, and -7), ultimately inducing neuronal nuclear DNA fragmentation and cytoskeleton disintegration, and leading to irreversible apoptotic cell death. In a rat middle cerebral artery occlusion/reperfusion (MCAO/R) model, CIRI-affected brain tissues show significantly increased cytoplasmic CytC release [15], and Apaf-1 expression and apoptosome formation are positively correlated with neuronal apoptosis rate [16]. Inhibiting this pathway can effectively reduce neuronal loss in the ischemic penumbra, confirming its core role in CIRI pathogenesis and providing a theoretical basis for targeted intervention to alleviate brain injury.

Astragaloside IV (AS-IV), the main active monomer of the traditional Chinese herb Astragalus membranaceus, exerts multiple pharmacological effects, including anti-inflammation, antioxidant, anti-apoptosis, and vascular endothelial protection, and shows promising application potential in treating cardiovascular and neurological diseases [17,18]. Recent studies have demonstrated that AS-IV alleviates CIRI by scavenging excessive reactive oxygen species (ROS) in brain tissue, downregulating pro-inflammatory factor (TNF-α, IL-6) expression, and inhibiting blood–brain barrier disruption. AS-IV also improves mitochondrial function, reduces mitochondrial membrane potential loss, and suppresses mitochondrial permeability transition pore (mPTP) opening in CIRI model rats. However, whether AS-IV exerts its anti-CIRI effect by regulating the CytC/Apaf-1 pathway to inhibit mitochondria-dependent neuronal apoptosis, and the underlying specific molecular mechanisms, remains unclear.

Based on the above pathological mechanisms and research gaps, this study established rat MCAO/R and cellular oxygen–glucose deprivation/reperfusion (OGD/R) models to simulate CIRI. Combined with molecular docking and molecular dynamics simulation to analyze the binding affinity of AS-IV with CytC/Apaf-1 pathway molecules (CytC, Apaf-1, caspase-9, and caspase-3), we detected neurological function, cerebral infarct volume, and neuronal apoptosis in AS-IV-treated rats, as well as the expression and activity of key pathway molecules in vitro and in vivo. This study systematically explored the neuroprotective effect of AS-IV against CIRI and its dependence on the CytC/Apaf-1 pathway, providing experimental and theoretical evidence for the clinical application of AS-IV in ischemic stroke.

## 2. Results

### 2.1. Results of Animal Experiments

#### 2.1.1. AS-IV Improves Neurological Function and Alleviates Brain Tissue Damage in MCAO/R Rats

**Figure 1:**
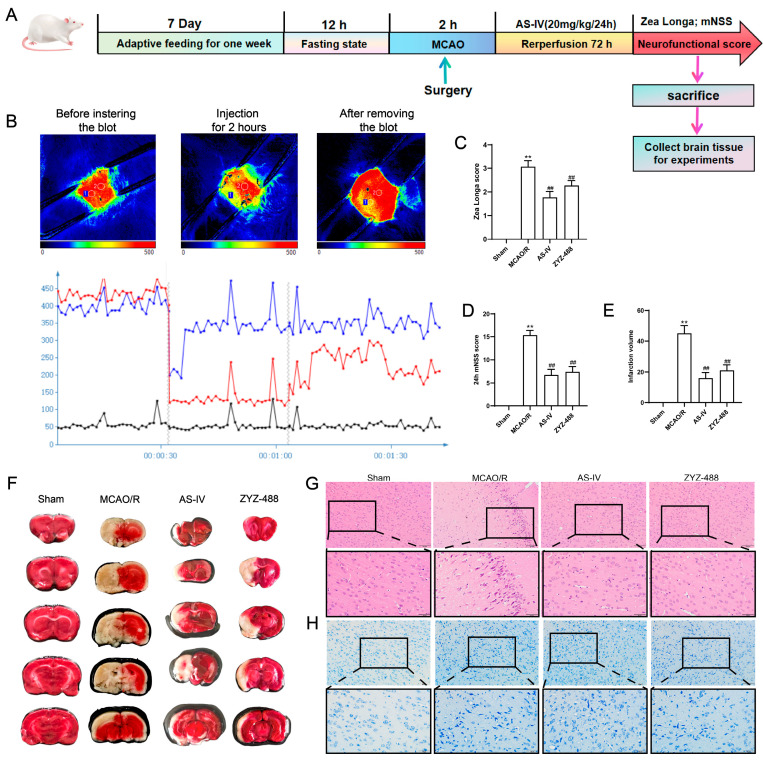
(**A**) In vivo experimental flowchart and modeling method. (**B**) Cerebral blood flow (CBF) by laser speckle imaging; Blue number 1= healthy side in CBF curves, red number 2 = affected side in CBF curves, The black lines represent the implanted blot. (**C**) Zea Longa score results. (**D**) Modified neurological severity score (mNSS) at 72 h. (**E**) Quantitative analysis of cerebral infarct volume. (**F**) TTC-stained brain sections: infarcted areas (white), non-infarcted areas (red). (**G**) HE staining (200×, 400×); black-bordered areas magnified to show tissue/cellular changes. (**H**) Nissl staining (200×, 400×); black-bordered areas magnified to show Nissl body changes. All experiments were performed with 3 independent biological replicates (*n* = 3). ** *p* < 0.01 vs. Sham group; ^##^ *p* < 0.01 vs. MCAO/R group.

To preliminarily elucidate the neuroprotective effects of AS-IV on cerebral ischemia–reperfusion injury (CIRI), this study systematically evaluated the neurological function, infarct volume, and histopathological changes in rats across all experimental groups. Figure 1A shows the experimental workflow: Rat underwent adaptive feeding and fasting, followed by middle cerebral artery occlusion (MCAO) surgery. Afterward, AS-IV intervention and reperfusion were performed, and subsequent neurological function scoring and tissue collection were conducted. Laser speckle imaging (Figure 1B) demonstrated that the cerebral blood flow (CBF) was stable in the Sham group. After MCAO, CBF significantly decreased, and it partially recovered after reperfusion. The Zea Longa score (Figure 1C) and mNSS Score (Figure 1D) indicated that the MCAO/R group had severe neurological deficits, while the scores were significantly reduced after treatment with AS-IV and ZYZ-488. TTC staining (Figure 1F) and quantification of infarct volume (Figure 1E) showed no infarction in the Sham group, whereas the MCAO/R group had extensive infarcted areas. The infarct volume was significantly reduced after drug treatment. HE staining (Figure 1G) and Nissl staining (Figure 1H) revealed normal neuronal and tissue morphology in the Sham group. In contrast, the MCAO/R group exhibited cellular edema, tissue disorganization, and pyknosis. These pathological changes were significantly alleviated after drug treatment.

#### 2.1.2. AS-IV Inhibits Neuronal Apoptosis and Regulates the Mitochondrial Apoptotic Pathway in MCAO/R Rats

**Figure 2:**
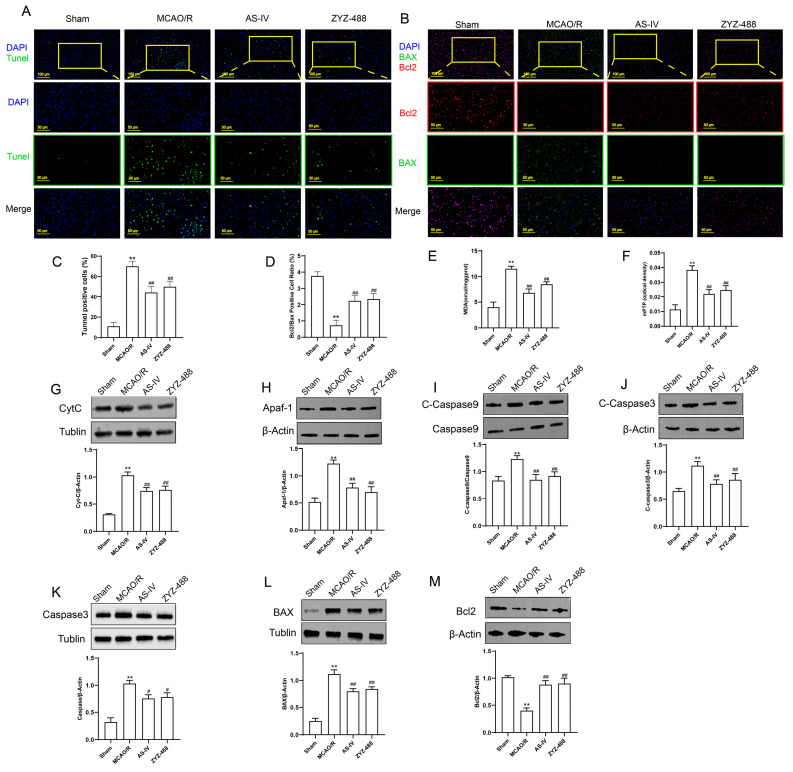
(**A**) Tunel staining to detect neuronal apoptosis. DAPI (blue) labels cell nuclei, TUNEL (green) labels apoptotic cells, and Merge represents merged images. (**B**) Immunofluorescence staining to detect the expression and distribution of Bcl2 (red) and BAX (green) (at ×200 and ×400 magnification). DAPI (blue) labels cell nuclei, and Merge represents merged images. (**C**,**D**) TUNEL-positive cell proportion and Bcl2/Bax positive cell ratio. (**E**,**F**) MDA levels and optical density (OD) of mPTP in each group. (**G**–**M**) Western blot results for detecting apoptosis-related proteins, including CytC, Apaf-1, Caspase9, C-Caspase9, Caspase3, C-Caspase3, BAX, and Bcl2, along with their quantitative analysis. All experiments were performed with 3 independent biological replicates (*n* = 3). ** *p* < 0.01 vs. Sham group; ^#^ *p* < 0.05, ^##^ *p* < 0.01 vs. MCAO/R group.

To further investigate the effects of AS-IV on apoptosis-related proteins, the expression levels of key pathway proteins were detected. Immunofluorescence, Western blot and TUNEL staining were used to observe AS-IV molecular effect on rat cerebral cortex (Figure 2). TUNEL (Figure 2A,C): The Sham group had few apoptotic cells; the MCAO/R group had more, which decreased after AS-IV/ZYZ-488 treatment. Immunofluorescence (Figure 2B,D): The Sham group had strong Bcl-2 (anti-apoptotic) and weak Bax (pro-apoptotic); MCAO/R showed the opposite, while treatment reversed this. MDA and mPTP (Figure 2E,F): Sham had low levels, while MCAO/R had high levels (mitochondrial damage); treatment improved. Western blot (Figure 2G–M): MCAO/R group had higher Cyt-C, Apaf-1, cleaved-Caspase-9/3, total Caspase-3 and Bax, lower Bcl-2; treatment regulated these. Conclusion: AS-IV/ZYZ-488 may inhibit MCAO/R-induced neuronal apoptosis via the mitochondrial apoptotic pathway.

To further explore the effects of AS-IV on apoptosis-related proteins, the expression levels of key proteins involved in the apoptotic pathway were determined. Immunofluorescence staining, Western blot analysis, and TUNEL staining were performed to evaluate the molecular effects of AS-IV in the rat cerebral cortex (Figure 2). As shown in TUNEL staining (Figure 2A,C), only a few apoptotic cells were observed in the Sham group, whereas the number of apoptotic cells was markedly increased in the MCAO/R group. Treatment with AS-IV or ZYZ-488 significantly reduced the number of apoptotic cells compared with the MCAO/R group. In immunofluorescence staining (Figure 2B,D), the Sham group exhibited strong expression of the anti-apoptotic protein Bcl-2 and weak expression of the pro-apoptotic protein Bax. In contrast, the MCAO/R group showed the opposite expression pattern, which was notably reversed by AS-IV or ZYZ-488 treatment. For MDA content and mPTP opening (Figure 2E,F), low levels were detected in the Sham group, while both MDA and mPTP were significantly elevated in the MCAO/R group, indicating severe mitochondrial damage. These indicators were effectively ameliorated after drug treatment. Western blot analysis (Figure 2G–M) demonstrated that the MCAO/R group exhibited significantly upregulated expression of Cyt-C, Apaf-1, cleaved-Caspase-9, cleaved-Caspase-3, total Caspase-3, and Bax, along with downregulated expression of Bcl-2. Treatment with AS-IV or ZYZ-488 significantly restored these protein expression levels. Collectively, these results suggest that AS-IV and ZYZ-488 may attenuate MCAO/R-induced neuronal apoptosis by regulating the mitochondrial apoptotic pathway.

### 2.2. Cell Experiment Results

#### 2.2.1. AS-IV Attenuates OGD/R-Induced Injury and Inhibits Apoptosis in PC12 Cells

**Figure 3:**
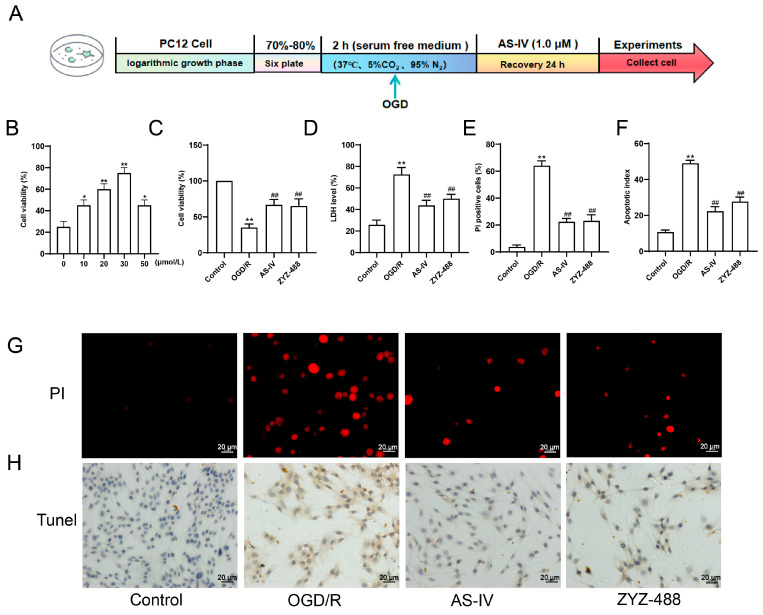
(**A**) Cell experimental flowchart. (**B**) Determination of YZYZ488 dose concentration. (**C**) Results of CCK-8 cell viability assay. (**D**) Results of LDH release level detection. (**E**) Statistical analysis of the proportion of PI-positive cells. (**F**) Statistical analysis of cell apoptosis index. (**G**) PI fluorescence staining results; red fluorescence indicates PI-positive dead cells. (**H**) TUNEL staining results: brown staining indicates apoptotic cells. All experiments were performed with 3 independent biological replicates (*n* = 3). * *p* < 0.05, ** *p* < 0.01 vs. control group; ^##^ *p* < 0.01 vs. ODG/R group.

To further explore the effect of AS-IV on cerebral ischemia–reperfusion injury (CIRI)-related cell apoptosis, we conducted in vitro verification. The results are as follows in Figure 3. Figure 3A shows the experimental workflow of OGD/R treatment and drug intervention on PC12 cells. Cell viability assay (Figure 3B,C) revealed that the optimal drug concentration of YZYZ488 is 30 μmol/L, and compared with the Control group, OGD/R treatment significantly decreased the viability of PC12 cells. After intervention with AS-IV or ZYZ-488, cell viability markedly increased. LDH release assay (Figure 3D) indicated that compared with the Control group, the LDH level in the OGD/R group was significantly elevated, reflecting aggravated cell damage. After treatment with AS-IV and ZYZ-488, the LDH level was significantly reduced, suggesting that the drugs alleviated cell damage. PI staining showed that compared with the Control group, the proportion of PI-positive cells in the OGD/R group increased significantly, while this proportion was notably reduced after treatment with AS-IV and ZYZ-488. TUNEL staining further demonstrated that compared with the Control group, OGD/R significantly induced an increase in PC12 cell apoptosis, whereas AS-IV and ZYZ-488 could effectively inhibit OGD/R-induced cell apoptosis (Figure 3E–H). In conclusion, AS-IV and ZYZ-488 can alleviate OGD/R-induced damage to PC12 cells, inhibit cell apoptosis, and improve cell viability.

#### 2.2.2. AS-IV Regulates the Mitochondrial Apoptotic Pathway in OGD/R-Injured PC12 Cells

**Figure 4:**
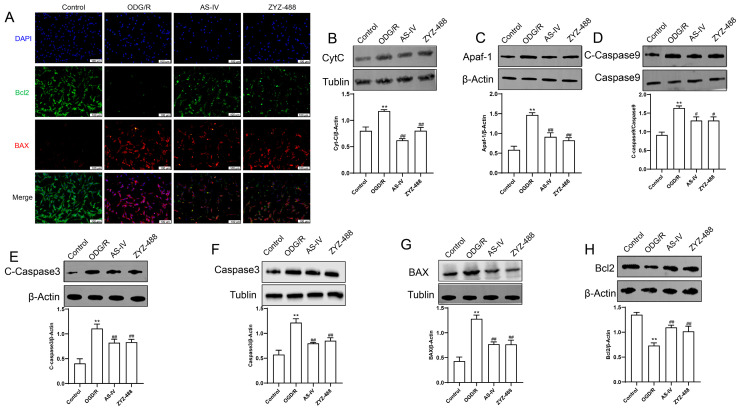
(**A**) Immunofluorescence staining to detect the expression and distribution of Bcl2 (green) and BAX (red) in cells of each group. DAPI (blue) labels cell nuclei, and Merge represents merged images. (**B**–**H**) Western blot results for detecting apoptosis-related proteins, including CytC, Apaf-1, Caspase9, C-Caspase9, Caspase3, C-Caspase3, BAX, and Bcl2, along with their quantitative analysis. All experiments were performed with 3 independent biological replicates (*n* = 3). ** *p* < 0.01 vs. Control group; ^#^ *p* < 0.05, ^##^ *p* < 0.01 vs. ODG/R group.

To further validate the regulatory effect of AS-IV on the mitochondrial apoptotic pathway observed in in vivo experiments at the cellular level, molecular detection techniques, including immunofluorescence and Western blot, were employed in this part of the study for mechanistic verification. In this part of the experiment, immunofluorescence and Western blot techniques were combined to systematically analyze the regulatory effects of different treatments on apoptosis-related molecules. Immunofluorescence results (Figure 4A) showed that in the OGD/R group, the fluorescence of the anti-apoptotic protein Bcl2 was significantly weakened, while the fluorescence of the pro-apoptotic protein BAX was obviously enhanced. However, treatment with AS-IV and ZYZ-488 could reverse this phenotype. Western blot analysis (Figure 4B–H) revealed that in the OGD/R group, the expression levels of upstream molecules of the mitochondrial apoptotic pathway (CytC, Apaf-1), downstream activated caspases (C-Caspase9, C-Caspase3), total Caspase3, and the pro-apoptotic protein BAX were all significantly increased; the expression of the anti-apoptotic protein Bcl2 was significantly decreased. Treatment with AS-IV and ZYZ-488 significantly downregulated the levels of the aforementioned pro-apoptotic molecules and upregulated the level of Bcl2. In conclusion, OGD/R treatment can activate the mitochondrial apoptotic pathway, while AS-IV and ZYZ-488 exert an anti-apoptotic effect by downregulating pro-apoptotic molecules (BAX, CytC, Apaf-1, C-Caspase9, and C-Caspase3) and upregulating the anti-apoptotic molecule (Bcl2).

### 2.3. Molecular Docking Validates the Direct Binding of AS-IV to Key Proteins in the Mitochondrial Apoptotic Pathway

**Figure 5:**
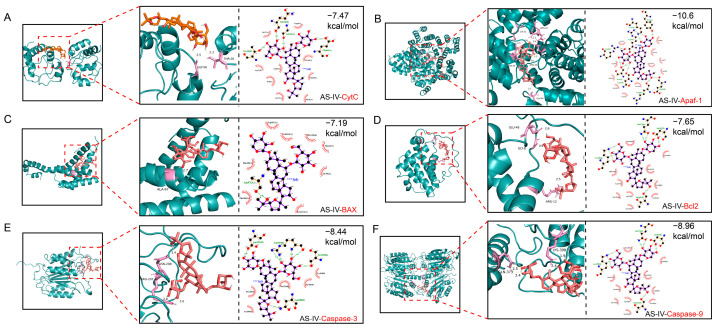
Molecular docking results of AS-IV with apoptosis-related proteins. (**A**–**F**) correspond to the following: CytC, Apaf-1, BAX, Bcl2, Caspase-3, and Caspase-9. Green represents protein, orange represents AS-IV.

To elucidate the molecular basis underlying the regulatory effect of AS-IV on the mitochondrial apoptotic pathway, we performed molecular docking assays to investigate its binding affinity and interaction modes with core proteins of this pathway. The results are shown in Figure 5. That AS-IV exhibited high affinity for all these six apoptotic proteins, with the following specific docking scores: CytC (−7.47 kcal/mol), Apaf-1 (−10.6 kcal/mol), BAX (−7.19 kcal/mol), Bcl2 (−7.65 kcal/mol), Caspase3 (−8.44 kcal/mol), and Caspase9 (−8.96 kcal/mol), as shown in Figure 5A–F. Through comparative analysis of docking scores and key intermolecular interactions with target-specific reference ligands, Apaf-1 was identified as AS-IV’s primary binding target with the most favorable binding characteristics (Figure 5B). Further study on intermolecular interactions revealed that the binding between AS-IV and target proteins was mainly mediated by non-covalent interactions, including conventional hydrogen bonds, carbon–hydrogen bonds, and π-alkyl interactions, with relevant results shown in Figure 5A–F.

### 2.4. Molecular Dynamics Simulation Confirms the Stable Binding of AS-IV to Mitochondrial Apoptotic Pathway Proteins

**Figure 6:**
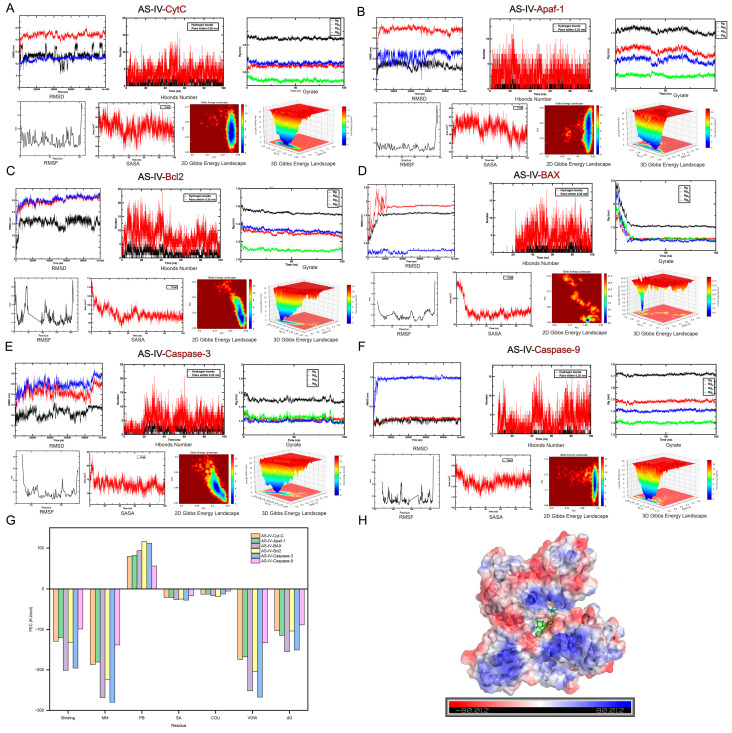
Molecular dynamics simulation analysis of the dynamic binding stability of AS-IV to mitochondrial apoptotic pathway key proteins. (**A**–**F**) Conformational dynamics and stability analysis of the complexes formed between AS-IV and CytC, Apaf-1, Bcl2, BAX, Caspase-3, and Caspase-9 during 100 ns MD simulations. RMSD (nm): Time evolution of root-mean-square deviation for the protein backbone (black), ligand (red), and complex (blue). Hbonds Number: Temporal profile of hydrogen bonds (black) and close contacts, reflecting persistent intermolecular interactions. Gyrate (nm): Radius of gyration (Rg) and its components (Rg_x_, Rg_γ_, Rg_z), indicating minimal overall protein compaction and structural integrity. RMSF (nm): Residue-wise root-mean-square fluctuation, highlighting flexible regions upon ligand binding. SASA (nm^2^): Solvent-accessible surface area over time, consistent with a stable hydrophobic core. 2D Gibbs Energy Landscape: Free energy projection onto the first two principal components (PC1, PC2), revealing a dominant, well-defined conformational basin. 3D Gibbs Energy Landscape: Free energy surface as a function of Rg (y) and RMSD (x), confirming a single, deep energy minimum corresponding to the stable complex conformation. (**G**) FEC analysis results; polar interactions (PB) serve as the main favorable force for the stable binding of AS-IV to target proteins. (**H**) Chemistic molecular dynamics simulation of the binding mode between AS-IV and Apaf-1. The Apaf-1 protein surface is electrostatically colored (red for negatively charged regions, blue for positively charged regions), clearly demonstrating the electrostatic interaction characteristics between AS-IV and the active pocket of Apaf-1. The AS-IV molecule is displayed as a ball-and-stick model (green), positioned within the key binding region of Apaf-1, intuitively illustrating its spatial binding pattern.

To further validate the dynamic stability of the binding between AS-IV and the key proteins of the mitochondrial apoptotic pathway identified by molecular docking, a 100 ns all-atom molecular dynamics simulation (MDS) was performed to investigate the structural flexibility of target proteins and the conformational stability of AS-IV-protein complexes under physiological conditions. After the simulation, systematic trajectory analysis was conducted using professional bioinformatics tools, and key structural and dynamic parameters, including root-mean-square deviation (RMSD), root-mean-square fluctuation (RMSF), hydrogen bond number, radius of gyration (Rg), solvent-accessible surface area (SASA), and free energy landscape (FEC), were calculated and analyzed (Figure 6). Figure 6A–F depict the dynamic behavior of AS-IV in complex with CytC, Apaf-1, Bcl2, BAX, caspase-3, and caspase-9, respectively. The RMSD plots indicate that all AS–protein complexes reached structural equilibrium within the simulation timescale, as evidenced by the stabilization of RMSD values after an initial equilibration phase. Concomitantly, the number of hydrogen bonds and radius of gyration remained relatively constant, suggesting that the binding of AS-IV did not induce significant global conformational changes in the target proteins. The RMSF profiles revealed localized flexibility in specific protein regions, while the SASA plots demonstrated a reduction in surface exposure upon ligand binding. The 2D and 3D Gibbs free energy landscapes further illustrated the conformational space and energy minima of each complex, indicating stable binding modes. Figure 6G presents the FEC for AS-IV with each target protein. The results show that the binding affinity is primarily driven by favorable van der Waals and electrostatic interactions, which are partially counterbalanced by the unfavorable polar solvation energy. The nonpolar solvation energy contributes favorably to the binding, consistent with the hydrophobic nature of the binding pockets. Figure 6H shows the electrostatic surface potential of the target protein in complex with AS-IV. The binding pocket is characterized by a complementary electrostatic potential, with regions of negative and positive charge facilitating the specific recognition and stable binding of AS-IV. In conclusion, the 100 ns MDS results fully confirmed that AS-IV could form stably bound complexes with the key proteins of the mitochondrial apoptotic pathway, with favorable intermolecular interaction forces and stable conformational characteristics, which provided a reliable dynamic molecular basis for the specific binding and regulatory effect of AS-IV on the mitochondrial apoptotic pathway.

## 3. Discussion

Currently, vascular recanalization has become the main clinical strategy for ischemic stroke, yet secondary brain injury induced by cerebral ischemic–reperfusion injury (CIRI) remains the core bottleneck limiting prognostic improvement in patients [19]. Clinical data demonstrate that 30–50% of patients receiving time-window recanalization therapy develop neurological deterioration due to CIRI, leading to permanent sequelae, including limb motor dysfunction and cognitive impairment, and even death in some cases from aggravated brain injury [20,21]. Despite recent progress in neuroprotective strategies targeting mitochondrial protection and apoptotic regulation [22,23,24], no effective systematic intervention regimen has been developed to date. Against this backdrop, the present study employed established middle cerebral artery occlusion/reperfusion (MCAO/R) rat and oxygen–glucose deprivation/reperfusion (OGD/R) cell models to investigate the effects and underlying mechanisms of astragaloside IV (AS-IV), with a specific focus on its neuroprotective mechanism via regulation of the mitochondrial apoptotic pathway, aiming to provide novel experimental evidence for addressing the current clinical predicament of ischemic stroke treatment.

Neuronal apoptosis is a core pathological process of CIRI-induced secondary brain injury, and its spatiotemporal dynamics directly modulate the progression and prognosis of brain damage [25]. Following cerebral ischemia–reperfusion, ischemia-hypoxia-induced cellular stress synergizes with reperfusion-triggered oxidative stress and mitophagy [26,27], jointly inducing apoptosis via multiple pathways, including the mitochondria-dependent and death receptor pathways [28]. Among these, massive neuronal apoptosis is the key driver of progressive neurological deficits [29,30]. As the central regulatory hub of apoptosis, mitochondrial functional integrity constitutes a core defense against apoptotic signaling [31]; mitochondrial damage leads to the release of pro-apoptotic factors such as CytC, which initiate downstream apoptotic cascade reactions [32] and act as a critical bridge linking mitochondrial impairment to neuronal apoptosis [33]. This renders the mitochondrial apoptotic pathway a prime therapeutic target for CIRI-related neuroprotection research [34,35]. In this study, we focused on the CytC/Apaf-1 pathway—a key regulatory node of the mitochondrial apoptotic pathway—and verified its central role in CIRI pathogenesis through in vivo and in vitro experiments, laying a solid foundation for exploring AS-IV’s intervention mechanism.

The CytC/Apaf-1 pathway is both a critical regulatory node of the mitochondrial apoptotic pathway [36] and a core mediator of CIRI-induced neuronal apoptosis [15]. Prior studies have confirmed that CIRI-induced ischemia-hypoxia reduces mitochondrial membrane potential and induces abnormal opening of the mitochondrial permeability transition pore (mPTP) [37], which promotes CytC release from mitochondria into the cytoplasm. CytC then binds to Apaf-1 and dATP to form the apoptosome, which activates the caspase-9/caspase-3 cascade reaction and ultimately triggers massive neuronal apoptosis, exacerbating brain injury [13]. Our in vivo and in vitro experiments further validated this pathway’s core role: in MCAO/R rats and OGD/R-injured PC12 cells, cytoplasmic CytC levels, Apaf-1 expression, and cleaved-caspase-9/3 activity were significantly elevated, and these indices were positively correlated with neuronal apoptosis rate and cerebral infarct volume. AS-IV intervention markedly inhibited the abnormal activation of key molecules in this pathway, concomitantly reducing neuronal apoptosis and alleviating brain injury. These results directly confirm that the CytC/Apaf-1 pathway is a key target for AS-IV to exert its anti-CIRI neuroprotective effect.

As the major active monomer of the traditional Chinese herb Astragalus membranaceus [38], AS-IV has attracted extensive research attention for its anti-CIRI properties [39]. Previous studies have shown that AS-IV mitigates oxidative stress- and inflammation-induced brain damage by scavenging reactive oxygen species (ROS) and downregulating pro-inflammatory factors, including TNF-α and IL-6 [40]; it also improves mitochondrial energy metabolism, increases ATP content, and restores mitochondrial membrane potential, thereby inhibiting the pathological cascade triggered by mitochondrial damage at the source [41]. Our prior work has additionally demonstrated that AS-IV exerts neuroprotection against CIRI by regulating autophagy and the calcium-sensing receptor pathway [40,42,43]. However, these studies focused on upstream pathological regulatory mechanisms and did not investigate the CytC/Apaf-1 pathway—the core executor of neuronal apoptosis—nor systematically verify AS-IV’s regulation of this key apoptotic pathway, with most research remaining at the level of modulating macroscopic pathological processes rather than targeting specific signaling pathways. The present study thus identifies a novel and complementary mechanism: AS-IV directly targets the CytC/Apaf-1 pathway to inhibit neuronal apoptosis, which distinguishes this work from our previous findings and further enriches the mechanistic network underlying AS-IV-mediated CIRI amelioration.

**Figure 7:**
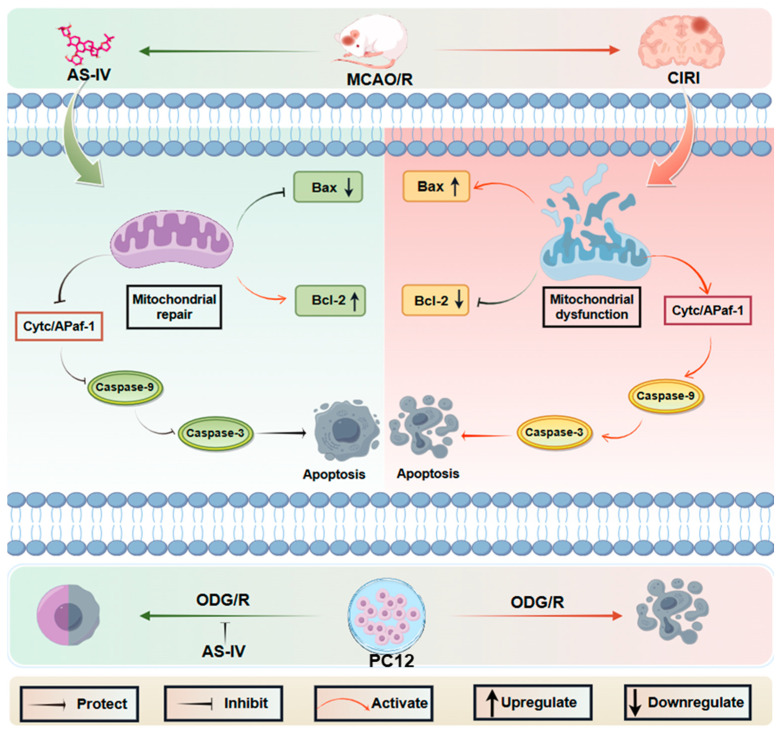
Mechanism summary (By Figdraw).

This study provides important supplements and novel insights to existing research on AS-IV and CIRI. First, molecular docking and molecular dynamics simulation confirmed that AS-IV stably binds to CytC/Apaf-1 pathway-related proteins, including CytC and Apaf-1, with the highest binding affinity for the Caspase Activation and Recruitment Domain (CARD) of Apaf-1, providing a theoretical basis for its direct targeted regulation of this pathway. Second, multi-dimensional experiments at the animal, cellular, and molecular levels clarified that AS-IV exerts anti-CIRI effects by directly targeting and inhibiting the CytC/Apaf-1 pathway, rather than merely indirectly regulating upstream pathological processes. Third, control experiments with the Apaf-1 inhibitor ZYZ-488 confirmed that AS-IV’s neuroprotective effect against CIRI is dependent on the inhibition of the CytC/Apaf-1 pathway, further verifying the specificity of its mechanism of action. Fourth, in vitro experiments confirmed that AS-IV protects OGD/R-injured PC12 cells, with its regulatory effect on the CytC/Apaf-1 pathway consistent with that observed in in vivo MCAO/R rat experiments (Figure 7). Collectively, these results provide more comprehensive and rigorous experimental evidence for the anti-CIRI mechanism of AS-IV, and lay a solid theoretical foundation for the subsequent development of AS-IV-based targeted therapeutic drugs for CIRI.

## 4. Materials and Methods

### 4.1. Main Reagents

Astragaloside IV (AS-IV) was procured from Shifeng Biotechnology Co., Ltd. (Shanghai, China), with a specification of 20 mg per vial, purity ≥ 98%, and Batch No.: 15082136. ZYZ-488 was supplied by MedChemExpress Biotechnology Co., Ltd. (Monmouth Junction, NJ, USA), under Batch No.: HY-100472. Antibodies were sourced from multiple vendors: The Apaf-1 antibody (Cat. No.: 8723) was obtained from Cell Signaling Technology, Inc. (Boston, MA, USA); CytC antibody (Cat. No.: 10993-1-AP) and caspase3 antibody (Cat. No.: 25128-1-AP) were both acquired from Wuhan Sanying Biotechnology Co., Ltd. (Wuhan, China); BAX antibody (Cat. No.: ab32503) and Bcl-2 antibody (Cat. No.: ab196495) were provided by Abcam plc. (Cambridge, UK). Servicebio Technology Co., Ltd. (Wuhan, China) supplied a range of reagents, including TTC staining solution (Cat. No.: G1017), phosphate-buffered saline (Cat. No.: G4202), HE staining kit (Cat. No.: G1005), rat anti-β-actin monoclonal antibody (Cat. No.: GB15001), and universal tissue fixative (Cat. No.: G1101). Other reagents and materials came from the following sources: a mitochondrial extraction kit was purchased from Beijing Sunshine Biotechnology Co., Ltd. (Beijing, China); mitochondrial permeability transition pore (mPTP) detection kit (Cat. No.: G10101) was procured from Shanghai Jinmei Gene Pharmaceutical Technology Co., Ltd. (Shanghai, China); the middle cerebral artery occlusion suture (Cat. No.: 2636-A3) was supplied by Beijing Xinnong Biotechnology Co., Ltd. (Beijing, China).

### 4.2. Animal Experiments

Sixty specific pathogen-free (SPF)-grade male Sprague–Dawley (SD) rats, with weights ranging from 260 to 280 g, were supplied by Beijing Vital River Laboratory Animal Technology Co., Ltd. (Beijing, China), holding Animal License No.: SCXK (Jing) 2022-0011. The experimental procedure received approval from the Ethics Committee of Hebei University of Chinese Medicine (Approval No.: DWLL202302022), and all operations were in strict compliance with the experimental animal ethics guidelines.

#### 4.2.1. Animal Grouping

Sixty male Sprague-Dawley (SD) rats were randomly divided into four groups: Sham operation group (Sham), model group (MCAO/R), Astragaloside IV group (AS-IV), and inhibitor group (ZYZ-488, an Apaf-1 inhibitor). Except for the Sham group, the other three groups all underwent MCAO/R modeling, among which the AS-IV group and the inhibitor group received drug intervention, respectively. The AS-IV group was given tail vein injection of AS-IV solution once every 24 h at the time of reperfusion and within the subsequent 72 h, with a dose of 20 mg/kg [40,42]. The ZYZ-488 group received tail vein injection of ZYZ-488 at the same time points and via the same route, with a dose of 30 mg/kg. The Sham group and MCAO/R group were given an equal volume of normal saline injection, and tissue samples were collected after 3 days of intervention.

#### 4.2.2. MCAO/R Animal Modeling Methodology

Rats were anesthetized with 1.5–2.5% isoflurane, placed supine, and cervical tissues dissected layer by layer to expose left CCA, ECA, and ICA (connective tissues separated to avoid injury). ECA was ligated, CCA/ICA clamped; silicone-coated nylon monofilament was inserted via ECA into ICA to MCA for focal ischemia. After 2 h, the monofilament was withdrawn for reperfusion; the ECA incision was ligated and wound was sutured. Sham group omitted monofilament insertion and ischemia–reperfusion. Rats were on 37 °C constant-temperature blanket during modeling.

#### 4.2.3. Detection of Cerebral Blood Flow Changes in Rats Using Laser Speckle Contrast Imaging

Rats were anesthetized; a longitudinal incision was made between the eye and ear, hook-fixed to expose the cerebral cortex, and the cerebral cortex was exposed. Bilateral symmetrical areas were selected. The laser speckle system lens was 14.0 cm from the area; blood flow data were collected at three stages: baseline, ischemia (2 h post-embolization), and reperfusion. Monitoring changes quantifies model validity and intervention effects.

#### 4.2.4. Evaluation of Neurological Deficit Severity in Rats Using the Zea Longa Neurological Deficit Scoring System

This study employed the Zea Longa scoring system to screen for neurological function. Rats with scores of 1–3 were randomly assigned to each experimental group, excluding rats with a score of 0 (no neurological deficit) and a score of 4 (moribund state). After establishing the MCAO/R model, neurological deficits were evaluated at 24 h post-reperfusion, and the scores of each group were recorded.

#### 4.2.5. Detection of Neurological Behavioral Impairment in Rats Using the Modified Neurological Severity Score (mNSS)

The Modified Neurological Severity Score (mNSS) was used to assess the neurological function of rats in each group at 24 h post-reperfusion, covering assessment dimensions of motor function, sensory function, balance, and reflex function. This scoring system has a range of 0–18 points, with higher scores indicating more severe neurological impairment. All scoring was performed independently by two researchers who were blinded to the experimental grouping to ensure the objectivity of the assessment results.

#### 4.2.6. Determination of Cerebral Infarct Volume in Rats Using TTC Staining

Rats were euthanized with excessive isoflurane 3 days post-reperfusion; brains were dissected immediately, rinsed with pre-cooled PBS to remove blood, and frozen at −20 °C for 30 min. Brains were cut into 2 mm coronal sections (from the olfactory bulb) with a brain mold, incubated with 2% TTC (37 °C, light-protected, 20 min, and flipped every 10 min), fixed with 4% paraformaldehyde for 24 h, and photographed. Cerebral infarct volume% was calculated via Image-Pro Plus 8.0.

#### 4.2.7. Observation of Histopathological Morphological Changes in Rat Cerebral Cortex Using HE Staining

After the rats were anesthetized and euthanized, brain tissues were dissected and fixed in 4% neutral formaldehyde for 48 h. Subsequently, the tissues were subjected to gradient ethanol dehydration, xylene clearing, and paraffin infiltration and embedding in sequence, then cut into 4 μm-thick sections using an ultramicrotome. After dewaxing and rehydration, the sections were stained with HE. They were then dehydrated with gradient ethanol, cleared with xylene, and finally mounted. Pathological images of the ischemic penumbra were collected under observation with a light microscope.

#### 4.2.8. Observation of Neuronal Injury in Rat Cerebral Cortex Using Nissl Staining

Cerebral cortex paraffin sections: Sequentially dewaxed with xylene, dehydrated with gradient alcohol. Stained with 1% toluidine blue, incubated at 60 °C for 30 min. Rinsed with distilled water 3× (3min), differentiated with 95% alcohol. After gradient alcohol dehydration and xylene clearing and mounted. Observed under inverted microscope (×200/×400) and photographed.

#### 4.2.9. Detection of Neuronal Apoptosis Level in Rat Cerebral Cortex Using TUNEL Staining

Paraffin sections: TdT + fluorescein-dUTP added, incubated 37 °C dark 60 min; citrate buffer-rinsed 3× (5min). DAPI added, incubated RT dark 10min; PBS-rinsed 2× (3min). Controls: Positive (DNase I 37 °C 30min) and negative (no TdT). Fluorescence microscopy (×200/400).

#### 4.2.10. Detection of Mitochondrial Permeability Transition Pore (mPTP) Opening Degree and Malondialdehyde (MDA) Changes in Rat Cerebral Cortex Tissue Using Kits

mPTP detection: Mitochondria extracted; BCA-adjusted to 10 mg/mL. A total of 20 μL suspension + 170 μL buffer (Reagent A) for 0 min absorbance; 10 μL swelling solution (Reagent B) added, RT absorbance recorded dynamically for 10 min. Result: 0–10 min absorbance difference (larger = more swelling). MDA detection: Mitochondria used and sampled per kit; 95 °C water bath 40 min, cooled, 3000× *g* centrifuged 10 min (supernatant). Distilled water zeroed, 532 nm absorbance measured; MDA activity calculated via formula.

#### 4.2.11. Detection of Positive Expression Rates of Bcl-2 and Bax Proteins in Rat Cerebral Cortex Using Immunofluorescence Staining

Dewaxed sections washed with water; 0.01 M sodium citrate (antigen retrieval) heated in microwave (medium power, 10 min), PBS-rinsed 3×. Blocked with 5% BSA (30 min), incubated with Bcl-2/Bax primary Ab (1:100) at 4 °C overnight. TBST-rinsed 3× (10 min×), and secondary Ab (Alexa Fluor 488/594, 1:500) incubated in dark (RT, 1 h). DAPI (1:1000) stained 5 min, PBS-rinsed 2×, mounted, and dried dark. Fluorescence microscopy captured images, which were then analyzed using Image-Pro.

#### 4.2.12. Detection of Protein Expression Levels of CytC, Apaf-1, Bcl-2, Bax, Cleaved-Caspase-3, Caspase-3, Cleaved-Caspase-9, and Caspase-9 in Rat Cerebral Cortex Using Western Blot

Cerebral cortex ischemic penumbra tissue proteins extracted; BCA assayed concentration. SDS-PAGE, membrane transfer, and blocked with non-fat milk. Primary Abs (β-actin 1:1000; CytC, Apaf-1 1:2000) incubated at 4 °C overnight. TBST-washed 3×, goat anti-rabbit secondary Ab (1:2500) incubated 2 h (RT). Washed and ECL-developed. Image-Pro measured gray values; relative expression vs. β-actin calculated.

### 4.3. Cellular Experiments

The rat adrenal pheochromocytoma cell line (PC12 cells) was purchased from BOSTER Company (Osaka, Japan). After being treated with nerve growth factor (NGF), the cells become highly differentiated and exhibit neuron-related characteristics.

#### 4.3.1. Cell Grouping and Modeling Method

PC12 cells were randomly divided into 4 groups: Normal Control Group (Control), Oxygen–Glucose Deprivation/Reperfusion Group (OGD/R, Model), AS-IV Group (100 μmol/L), and ZYZ-488 Group (30 μmol/L). The Control group was cultured normally in complete DMEM medium in a CO_2_ incubator. Cells in the other groups were used to establish the OGD/R cell model: complete DMEM medium was replaced with glucose-free EBSS, and the cells were incubated in a tri-gas incubator(Thermo Fisher, Waltham, MA, USA) (94% N_2_, 5% CO_2_, 1% O_2_) for 2 h (2 h of OGD). After OGD, the Model group was refed with complete DMEM medium: the AS-IV group and ZYZ-488 group were refed with DMEM medium containing Astragaloside IV (AS-IV, 100 μmol/L) [43] and DMEM medium containing ZYZ-488 (30 μmol/L), respectively. All groups were then placed in a CO_2_ incubator for further culture for 24 h (24 h of reoxygenation and re-glucosation).

#### 4.3.2. Detection of PC12 Cell Viability in Each Group Using the CCK-8 Assay

Cells in each group were seeded into 96-well plates at a density of 1 × 10^5^ cells/mL. Then, 10 μL of CCK-8 solution was added to each well, gently pipetted to mix well, and incubated in a CO_2_ incubator for 50 min. A microplate reader was used to measure the absorbance (OD value) of each well at a wavelength of 450 nm. A higher OD value indicates stronger cell viability. The calculation formula for cell survival rate is as follows: [(OD value of experimental group − OD value of blank group)/(OD value of control group − OD value of blank group)] × 100%.

#### 4.3.3. Determination of Lactate Dehydrogenase (LDH) Leakage Rate in PC12 Cells of Each Group Using the LDH Assay

Grouped cell supernatants + LDH reagent in 96-well plates, mixed, incubated 5min (RT); OD450 measured (supernatant LDH). 1% Triton X-100 added to original plates, lysed 20 min (RT); culture solution + LDH reagent incubated 5 min (RT), OD450 measured. LDH leakage rate (%) = [supernatant LDH/(supernatant + lysate LDH)] × 100%.

#### 4.3.4. Evaluation of PC12 Cell Membrane Damage in Each Group Using PI Staining

Polylysine-coated slides in 12-well plates; PC12 cells seeded (1 × 10^5^ cells/mL, monolayer). Fresh, light-protected PI working solution (20 μg/mL PBS) prepared. Cell climbing slices rinsed with PBS (1 min × 3), incubated with PI (37 °C, dark, 30 min), rinsed again (5 min × 3). Slightly dried and mounted; observed under fluorescence microscope (six random fields, PI-positive: red fluorescence). PI-positive rate (%) = (Positive cells/Total cells) × 100%.

#### 4.3.5. Detection of Apoptotic Index in PC12 Cells of Each Group Using TUNEL Staining

Polylysine-coated slides in 12-well plates; PC12 cells seeded (1 × 10^5^ cells/mL, monolayer); fixed with 4% paraformaldehyde (30 min) and PBS-rinsed (3 min × 3). A total of 0.2% Triton X-100 (RT, 20 min) and H_2_O_2_-treated (RT, 5 min). TUNEL mix (TdT:dUTP = 1:9; Control: dUTP), incubated (37 °C, dark, 60 min, humidified), and rinsed. Converter-POD (30 min, same conditions) and rinsed. DAB (RT, dark, 10 min), rinsed. Counterstained, dehydrated, cleared, mounted (positive nuclei: brown-yellow). Three fields/group observed; apoptotic index = Positive/Total cells.

#### 4.3.6. Detection of Apoptotic Proteins Bcl-2 and Bax Expression in PC12 Cells of Each Group Using Immunofluorescence

Cell climbing slices: Fixed with 4% paraformaldehyde (30 min), air-dried (5 min), and PBS-rinsed (2 min × 3). A total of 0.2% Triton X-100 (20 min), PBS-rinsed again. A total of 4% BSA blocked (30 min). Bax/Bcl-2 primary Ab (1:100) incubated at 4 °C overnight. Washed, secondary Ab (1:300/1:200) incubated in dark (60 min). Washed, DAPI-stained (10 min), and PBS-rinsed. Mounted; three fields/group observed.

#### 4.3.7. Detection of Mitochondrial Apoptotic Pathway Proteins (CytC, Apaf-1, Bcl-2, Bax, Cleaved-Caspase-3, Caspase-3, Cleaved-Caspase-9, and Caspase-9) Expression in PC12 Cells Using Western Blot

PC12 cells were seeded in 6-well plates; total proteins were extracted with lysis buffer. SDS-PAGE, membrane transfer and blocked with non-fat milk. Primary Abs (β-actin 1:1000; CytC, Apaf-1, Caspase-3/9 1:2000; Bcl-2 1:3000; Bax 1:1000; Cleaved-Caspase-3 1:1500, Cleaved-Caspase-9 1:2000) incubated overnight. Washed, secondary Ab (1:8000) incubated 1 h (RT); ECL, imaged. Bands analyzed via Image-Pro Plus (relative to β-actin).

### 4.4. Molecular Docking

To predict the binding energy between astragaloside IV and related proteins (CytC, Apaf-1, Bcl-2, Bax, Caspase-3, and Caspase-9), molecular docking and molecular dynamics simulation were used. The detailed PDB structural information of the six target proteins is as follows: CytC (PDB: 5O10, 1.36 Å resolution, Homo sapiens), Apaf-1 (PDB: 4RHW, 2.1 Å resolution, Homo sapiens), BAX (PDB: 4BD6, 2.494 Å resolution, Homo sapiens), Bcl-2 (PDB: 9EW8, 1.488Å resolution, Homo sapiens), Caspase3 (PDB: 1GFW, 2.8 Å resolution, Homo sapiens), Caspase9 (PDB: 2AR9, 2.8 Å resolution, Homo sapiens). For molecular docking, AutoDockTools 1.5.6 and AutoDock Vina 1.2 were employed: ligand structures from PubChem were energy-minimized via ChemOffice 14.0, converted to PDBQT format with hydrogen addition using AutoDockTools; target crystal structures from RCSB PDB were processed with PyMOL 4.5.2 (solvent removal, hydrogen addition, binding pocket definition). Site-specific PDBQT grids generated by AutoDockTools enabled semi-flexible docking via AutoDock Vina 4.2. Binding affinity was quantified by binding free energy calculation, and final binding modes/interactions were visualized using PyMOL.

### 4.5. Molecular Dynamics Simulations

Molecular dynamics simulations (MDS) were conducted with GROMACS 2022.3. Small molecules were preprocessed with GAFF (AmberTools 22); Gaussian 16W was used for hydrogenation and restrained electrostatic potential (RESP) calculation, with data integrated into the MDS system’s topological file. Simulations were run at 300 K and 1 bar using the AMBER99SB-ILDN force field, with TIP3P water as solvent and Na^+^ ions for charge neutralization. Energy minimization was performed via the steepest descent method, followed by 100,000-step NVT and NPT equilibration. Unrestrained MDS was run for 5,000,000 steps (2 fs/step), totaling 100 ns. Trajectories were analyzed with GROMACS built-in tools, calculating profiles including RMSD, RMSF, radius of gyration, SASA, and FEC [44].

### 4.6. Statistical Methods

Data are expressed as mean ± standard deviation (SD). Statistical analyses and figure generation were conducted using GraphPad Prism software (version 8.0, San Diego, CA, USA). For normally distributed data, analysis of variance (ANOVA) was performed. If variance homogeneity was satisfied, a *t*-test was applied; otherwise, the Dunnett test was used. When data did not conform to a normal distribution, non-parametric tests were conducted. A *p*-value < 0.05 was considered statistically significant.

## 5. Conclusions

In conclusion, the results of this study confirm that astragaloside IV (AS-IV) can alleviate CIRI by directly targeting and inhibiting the CytC/Apaf-1 mitochondrial apoptotic pathway, while simultaneously repairing mitochondrial function and regulating the expression of apoptosis-related proteins. This conclusion not only clearly clarifies the specific mechanism of AS-IV in the targeted regulation of the core apoptotic pathway in CIRI, addressing the limitation of insufficient elaboration on its molecular mechanism for regulating this pathway in previous studies, but also provides solid experimental data support and a theoretical basis for the development of AS-IV as a potential candidate drug for the clinical treatment of ischemic stroke.

## 6. Limitations

Limitations of cell models: PC12 cells are widely used as a preclinical alternative model for cerebral ischemia and neuroprotection research. Under the induction of nerve growth factor (NGF), these cells can differentiate into neuron-like phenotypes, express neuronal markers, and secrete neurotransmitters, with extensive research supporting their applications. However, the NGF-induced differentiation of PC12 cells (derived from rat adrenal pheochromocytoma) used in this study still exhibits differences in physiological characteristics and signaling pathway activation patterns compared to primary brain neurons, and cannot fully simulate the true pathological responses of primary neurons under CIRI conditions. Narrow observation time window: This study only monitored indicators within 72 h post-reperfusion in rats, without exploring the long-term protective effects of AS-IV on CIRI or the safety of long-term administration. While AS-IV was shown to modulate the expression and activity of key CytC/Apaf-1 pathway proteins, no genetic knockdown/knockout experiments (e.g., Apaf-1 siRNA in PC12 cells) were conducted to directly confirm that its neuroprotective effect is specifically dependent on this pathway. Although ZYZ-488 as a comparator partially verifies the link between AS-IV’s efficacy and CytC/Apaf-1 pathway inhibition, the absence of pathway-specific genetic evidence weakens the robustness of our mechanistic conclusions; supplementary genetic manipulation experiments would substantially strengthen the causal association between AS-IV-mediated CytC/Apaf-1 pathway regulation and its anti-CIRI effects.

## Data Availability

The original contributions presented in this study are included in the article. Further inquiries can be directed to the corresponding authors.

## Abbreviations

The following abbreviations are used in this manuscript:

Apaf-1	Apoptosis protease activating factor-1
AS-IV	Astragaloside IV
CBF	Cerebral blood flow
CCA	Common carotid artery
CIRI	cerebral ischemia–reperfusion injury
CytC	Cytochrome c
ECA	External carotid artery
FEC	Free energy landscape
ICA	Internal carotid artery
MCAO/R	Model of middle cerebral artery occlusion/reperfusion
MDS	Molecular dynamics simulation
mNSS	Modified neurological severity score
mPTP	Mitochondrial permeability transition pore
OGD/R	Oxygen–glucose deprivation/reperfusion
Rg	Radius of gyration
RMSD	Root-mean-square deviation
RMSF	Root-mean-square fluctuation
SASA	Solvent-accessible surface area
TTC	2,3,5-Triphenyltetrazolium Chloride
TUNEL	Terminal deoxynucleotidyl transferase-mediated dUTP nick end labeling
ZYZ-488	Apaf-1 inhibitor
